# Berlin

**Published:** 1855-06

**Authors:** 


### BERLIN.

The following is the Report of Meteorology and Epidemics in Berlin, during the winter months of January and February, 1855.

*January.* Up to the 12th of the month, the weather was just the same as in the latter half of December. The temperature of the air increased remarkably between the 5th and the 9th; but rain, snow, and a stormy west wind were the prevailing phenomena. The 5th, 7th, and 8th, were the only days on which a very cloudy sky was unaccompanied by rain and snow. Frost took place in the nights of the 3rd and 10th: but on the 13th it set in permanently, and remained till the end of the month. The temperature of the air in this period diminished much, and varied principally between the 17th and 23rd. The wind was northerly on the 13th, 15th, 16th, and 31st; westerly on the 14th, and from the 26th to the 29th; easterly from the 17th to the 23rd, and on the 30th; and southerly on the 24th and 25th. The wind was most violent on the 14th and 20th. Snow fell daily, but not in remarkable quantity, during the latter half of the month, except on the 16th, 18th, 19th, 22nd, 23rd, 30th, and 31st. The only perfectly clear days were the 19th and 31st. Fog was present on the mornings of the 18th and 23rd, and during the whole day on the 22nd.

The following variations in temperature were observed:—

| | | | | | | | | |
|---|---|---|---|---|---|---|---|---|
| At 7 a.m. | from | 42°. 8F. | on | the 6th, | to | 5°F. | on | the 31st. |
| At 2 p.m. | „ | 43°. 7F. | „ | 6th, | „ | 14°F. | „ | 18th. |
| At 9 p.m. | „ | 43°. 7F. | „ | 7th, | „ | 5°F. | „ | 18th. |

The average temperature in the morning was 24°. 1F.; in mid-day 27°.5, and in the evening 25°.F. The highest daily temperature was observed on the 8th; the lowest on the 18th. The barometer was generally high: it varied chiefly within the first ten days. Its readings were 346.07 Paris lines on the 10th, and 327.39 on the 1st. The average readings were, at 7 a.m. 337.8 Paris lines; at 2 p.m., 337.76; and at 9 p.m. 338.14.

The wet weather with which the month commenced, ceased at the end of the first week, and was succeeded by dry cold, which was at times rendered almost insupportable by the high east and north winds. The change of weather was followed by a change in the form of disease. The great number of catarrhal affections, which had been the prevailing complaints in the early part of the winter, diminished more and more; and, with the exception of unimportant cases of irritation of the eyelids, nose, and fauces, there was certainly a very great decrease in these disorders. Inflammatory diseases of the lungs were not in the proportion which might be expected from the state of the weather; for while the cold, the dryness of the air, and the direction and violence of the wind were very favourable to these diseases, yet much fewer persons suffered from them than at other times, as often in March and April, when the atmospheric conditions alluded to are much less intense. In chronic diseases of the respiratory organs, especially in consumption, the mischievous influence of the weather was much more remarkable; not only was hæmoptysis more frequent and violent, but the cases proved fatal at a more early period than usual. Acute rheumatism, of various degrees of intensity, was frequent. Toothache and earache were especially prevalent. Erysipelatous inflammation of the skin was among the more frequent diseases. Measles and scarlatina were rare. At the beginning of the month, cases of gastric and gastro-nervous fever occurred more frequently than in December; but they decreased remarkably in the latter half of January. In connection with the change of weather, the frequency and severity of cases of diarrhœa was remarkable. In the middle of the month a case of cholera occurred in the practice of Dr. Wolff, which, from the rapidity of its development, and the high stage which it reached, might be compared with the more severe cases of the epidemic form of this disease. Intermittent fever had not diminished in the number of cases. This is to be attributed to imperfect protection against the weather, insufficient clothing, bad condition of dwellings, and insufficiency of food, in many persons, which were the cause of many relapses of this disease, and of the occurrence of sequelæ, especially dropsy. The prevailing type was quartan.

Among domestic animals, especially horses, there were not many cases of disease. The prevailing type of disease among horses was catarrho-rheumatic, and was mostly manifested in the form of pulmonary catarrh, acute rheumatism, and even inflammation of the lungs and pleura. Here and there were cases of influenza, induration of the liver, rheumatic colic and enteritis, and a few cases of tetanus, gastritis, glandular swellings, rot, and worms. Among oxen, the prevalent diseases were acute rheumatism, suppurative inflammation, and gastro-rheumatic fever. Many cows calved prematurely. In scattered places there were cases of pleuro-pneumonia. Dogs suffered from catarrh, rheumatism, cough, water in the chest, convulsions, paralysis, and cutaneous eruptions. No cases of rabies were observed.

*February.*—Some rain fell on the 4th, 5th, and 25th; and snow on the 6th, 11th, 13th, 15th, 19th, and 21st. The warmest days were the 2nd, 9th, 10th, 24th, and 27th; in the remaining time a hazy or overcast sky prevailed, although there was some alternation of sunshine and starlight nights. Fogs were present on the mornings of the 17th, 22nd, and 26th. With slight variations, the north wind prevailed on the 1st, 2nd, 15th, and 27th; the east on the 3rd, from 7th to 14th, and from 20th to 22nd; the south wind from the 4th to the 6th, and the west wind from the 16th to the 19th, from 23rd to 26th, and on the 28th.

The following variations of temperature occurred during the month:—

| | | | | | | | | |
|---|---|---|---|---|---|---|---|---|
| 7 p.m. | from | 29°.75F. | on the | 26th | to | —7°5.F. | on the | 10th. |
| 2 p.m. | ,, | 37°.5F. | ,, | 26th | ,, | 3°.5F. | ,, | 10th. |
| 9 p.m. | ,, | 34°.75. | ,, | 25th | ,, | 0°.15F. | ,, | 10th. |

The average temperature was, in the morning, 14°.6F.; at mid-day, 21°.9F.; and in the evening, 16°.87F.

The weather in February agreed with that of the preceding month, in so far that the number of cold windy days far exceeded those of the calm days, on which the cold was less intensely felt. The forms of disease whose origin was to be traced to the influence of the weather, were the same as prevailed during January; such as inflammatory rheumatism, and affections of the organs of respiration. The first, which, without doubt, could be ascribed to the unavoidable chilling and wetting of the feet in the snow, in this month, chiefly affected the joints; the latter assumed the same forms as in January. The frequency of inflammation of the lungs in this month was not in direct ratio to the weather, whereas catarrhal bronchitis had begun to increase. As the cold abated a catarrhal fever appeared; the local symptoms of which were catarrh of the nose, eyes, and fauces; the general symptoms being fever, lasting one, two, or more days, and great weakness of the limbs. The most permanent symptom was a violent and frequent cough, which was kept up day and night by irritation of the glottis. Hoarseness was a very common complication of this catarrhal fever; the sudden and extensive spread of which showed it to be epidemic. Its origin from contagion was proved by the fact that, as a rule, the disease spread rapidly from one member of a family to the others. Gastric and gastro-nervous fevers occurred in about the same numbers; and their severity and influence on life maintained about the same proportions as in January. Intermittent fever continued to about the same extent; but the tertian type was manifested in several cases. Puerperal fever had again begun to spread. Measles rather increased in frequency; scarlatina broke out here and there, sometimes in so malignant a form as would not be expected in the time of the year and state of the weather; erysipelas, especially of the face, appeared in a pretty large number of cases. The prolonged duration of the winter's cold had the same, if not a more deadly influence than that in January on persons weakened by age and sickness.

The state of health of domestic animals was, on the whole, tolerably good in February. The diseases most frequently observed among horses were rheumatism, and catarrh, with here and there quinsy, pleurisy and pneumonia, catarrhal and rheumatic ophthalmia, rheumatic colic and intestinal inflammation, gastritis, principally of an asthenic character, diarrhœa, inflammation of the brain, tetanus, malignant swellings, glanders and worms. Many cows suffered from rheumatic affections: there were also many cases of abortion and diarrhœa. Pleuropneumonia was confined to one locality; and some cases of true cow-pock were found in one stall. Among sheep and pigs, disease appeared here and there in different places. Dogs suffered moderately, frequently from cough; some had pneumonia, acute rheumatism, and gastritis; a few had cramps. No cases of rabies were noticed.

